# Determinants of nutritional status among children under age 5 in Ethiopia: further analysis of the 2016 Ethiopia demographic and health survey

**DOI:** 10.1186/s12992-019-0505-7

**Published:** 2019-11-06

**Authors:** Zerihun Yohannes Amare, Mossa Endris Ahmed, Adey Belete Mehari

**Affiliations:** Institute of Disaster Risk Management and Food Security Studies, Bahir Dar University, Bahir Dar, Ethiopia

**Keywords:** Stunting, Wasting, Children under age 5, Demographic and health survey, Ethiopia

## Abstract

**Background:**

The aim of this study was to examine the determinants of nutritional status among children under age 5 (0–59 months) in Ethiopia. Child malnutrition is an underlying cause of almost half (45%) of child deaths, particularly in low socioeconomic communities of developing countries. In Ethiopia, the prevalence of stunting decreased from 47% in 2005 to 39% in 2016, but the prevalence of wasting changed little over the same time period (from 11 to 10%). Despite improvements in reducing the prevalence of malnutrition, the current rate of progress is not fast enough to reach the World Health Organization global target for reducing malnutrition 40% by 2025.

**Methods:**

This study used data from the 2016 Ethiopia Demographic and Heath Survey (EDHS). The analysis used stunting and wasting as dependent variables, while the independent variables were characteristics of children, mothers, and households. Logistic regression was used to analyze the determinants of nutritional status among children. Bivariate analysis was also used to analyze the association between the dependent and independent variables.

**Results:**

Study results show that child’s age, sex, and perceived birth weight, mother’s educational status, body mass index (BMI), and maternal stature, region, wealth quintile, type of toilet facility, and type of cooking fuel had significant associations with stunting. Child’s age, sex, and perceived birth weight, mother’s BMI, and residence and region showed significant associations with wasting. The study found that child, maternal, and household characteristics were significantly associated with stunting and wasting among children under age 5.

**Conclusion:**

These findings imply that a multi-sectorial and multidimensional approach is important to address malnutrition in Ethiopia. The education sector should promote reduction of cultural and gender barriers that contribute to childhood malnutrition. The health sector should encourage positive behaviors toward childcare and infant feeding practices. More should be done to help households adopt improved types of toilet facilities and modern types of cooking fuels.

## Introduction

Malnutrition during childhood is the outcome of insufficient food intake, diarrhea and other infections, lack of sanitation, and low parental education [1, 2]. Poor diets and disease are due to food insecurity, inadequate maternal and child care, and poor health services and environment [3]. These factors cause measurable adverse effects on body function and clinical outcome [4]. This problem leads to most of the anthropometric deficits found among children under age 5 in the world’s least developed countries [5]. Despite existing interventions to address child malnutrition, it is still a major global public health problem [6]. Child malnutrition is an underlying cause for almost half (45%) of child deaths, particularly in low socioeconomic communities of developing countries [7]. Globally in 2018, an estimated 149 million children under age 5 were stunted and 49 million children were wasted [8].

In sub-Saharan Africa, the prevalence of stunting is declining but remains over 30% [8]. Among sub-Saharan countries, the prevalence of wasting in Ethiopia is 10%, and the prevalence of stunting is 38% [9]. Within Ethiopia, there is a regional variation in stunting and wasting. Amhara, Benishangul-Gumuz, Afar, and Dire Dawa are the most affected by child stunting (41–46%), whereas wasting is highest in Somali (23%), Afar (18%), and Gambella (14%) [9]. The reason may be hard-to-reach areas of Afar, Somali, Benishangul-Gumuz, and Gambella were characterized by drought-related public health and nutrition problems [10]. The current rate of progress is not fast enough to reach the World Health Organization (WHO) global target of a 40% reduction in the number of stunted children by 2025 [11].

Thus to achieve this global target for 2025 in Ethiopia, a situational analysis is required to determine how many children under age 5 are stunted and wasted and to assess key determinants of malnutrition in specific social and geographical locations [11]. Among the factors that cause child malnutrition in Ethiopia are insufficient availability of food, inadequate provision of a healthy environment, such as sanitation and hygiene, women’s status concerning decision-making power, and factors related to political economy [12, 13]. Some studies have linked low birth weight, maternal short stature, household wealth quintile, place of residence, region, mother’s education, child’s age and sex, and perceived birth size to children’s nutritional status [14, 15]. However, other studies [16–18] have found consistent disparities in the prevalence of child malnutrition in terms of children’s age, sex, and birth size.

At the policy and program level, Ethiopia has many strategies and programs to reduce levels of malnutrition as part of its national development agenda. Some of the major strategies and programs include: the growth and transformation plan (GTP), National Nutrition Plan (NNP), the Seqota Declaration (SD), National Food Security Strategy, Nutrition Sensitive agriculture (NSa) strategy, school health and Nutrition Strategy (ShNS), the Productive Safety Net Program (PSNP), and Food Safety and Quality related regulatory activities [19]. The Seqota Declaration adopted Sustainable Development Goal 2 (SDG2), with the aim to end hunger, achieve food security, and improve nutrition, and promote sustainable agriculture by 2030 [20].

The Government of Ethiopia through the Ministry of Health also launched the health extension program in 2003 to achieve the country’s progress in meeting the Millennium Development Goals (MDGs). The health extension workers are mainly to improve access to care in rural communities. They spend 15% of their time with infants and children under age 5 [21]. All of these listed efforts have brought positive impact in improving food and nutrition security [19]. However, there was a fragmented approach leading to inadequate multi-sectorial coordination in responding to food and nutrition demand. As a result, severe malnutrition has remained a serious challenge [19]. Thus, this finding will be used as a source of information to implement the newly introduced national food and nutrition policy. Understanding the extent and the causes of the problem will allow for appropriate planning and interventions. This study will help health practitioners and other policymakers to enable a multi-sectoral response to malnutrition by identifying its determinants among children under age 5. This is important to achieving the Sustainable Development Goals and to meeting the global target by 2025.

## Methodology

### Data

This study used data from the 2016 Ethiopia Demographic and Health Survey (EDHS), which was conducted by the Central Statistical Agency (CSA) of Ethiopia, with technical support from ICF. The authors have got permission from ICF-DHS program to use the EDHS data and accessed through https://www.dhsprogram.com/data/dataset_admin/login_main.cfm‘.

The EDHS is a population-based household survey designed to provide representative data for the country as a whole and for nine regional states and two city administrations of Ethiopia. This study considered live children age 0–59 months with anthropometry data in the analysis of determinants of nutritional status among children under age 5 in Ethiopia. Missing values in the 2016 EDHS dataset were not included in the analyses. Not-alive children (635) and children with no anthropometry data from stunting condition (1000) or wasting condition (927) were also omitted from the analysis.

### Variables

#### Dependent variables

The three anthropometric indicators mostly used for monitoring malnutrition in children are: stunting (low height-for-age); underweight (low weight-for-age); and wasting (low weight-for height). However, underweight is the composite of stunting and wasting, so this study used stunting and wasting for the analysis. They were defined using the WHO child growth standards. Stunting and wasting were coded as binary variables based on the standard definitions.

#### Independent variables

To analyze the determinants of nutritional status among children under age 5, the study considered the following characteristics as independent variables: 1) child characteristics, including age (less than 6 months, 6–11 months, 12–17 months, 18–23 months, and 24–59 months), sex (male, female), birth order (first, 2–4, 5 or higher), and perceived birth weight (very small, smaller than average, average, and very large or larger than average); 2) maternal characteristics, including marital status (never married, married, widowed/divorced/separated), age at child’s birth (12-19, 20-49), educational status (no education, primary, secondary and above), working status (yes, no), body mass index (underweight, normal, overweight), and maternal stature (normal stature, short stature); 3) household characteristics, including place of residence (urban, rural), region (nine regions and two city administrations), wealth quintile (poorest, poorer, middle, rich, and richest), drinking water source (improved, non-improved), toilet type (improved, non-improved), and cooking fuel type (modern, traditional).

In the DHS, wealth quintile is calculated as an index based on consumer goods such as television, bicycle, or car. Household characteristics such as toilet facility, source of drinking water, and flooring materials are also considered in calculating the wealth index. These scores were derived using principal component analysis. Based on the 2016 EDHS, the water sources and toilet facilities were recoded as either improved or non-improved. Piped water, public taps, standpipes, tube wells, boreholes, protected dug wells and springs, and rainwater were considered as improved water sources. Non-shared toilet of the following types: flush/pour flush toilets to piped sewer systems, septic tanks, and pit latrines; ventilated improved pit (VIP) latrines; pit latrines with slabs. Composting toilets were considered as improved toilet facilities for this analysis. For household fuel type, electricity, natural gas, biogas, and kerosene were categorized as modern fuel. Charcoal, wood, animal dung, and other agricultural crops and straw were considered as traditional fuel.

### Statistical analysis

Descriptive statistics were used to describe and illustrate the background characteristics of children age 0–59 months. Stata 15.1 version was used to analyze the data. Sample weights were applied in all analysis due to the two-stage cluster sampling design in the EDHS dataset. Multicollinearity among independent variables was checked using variance inflation factors (VIF). Due to multicollinearity, preceding birth interval was removed from the analysis. Multiple logistic regression was used to determine the associations between predictors and nutritional outcomes after adjusting for other covariates. The study also used Pearson chi-square test for bivariate analysis.

## Results

As Table 1 shows, the majority of children (61%) were age 24–59 months, more than half (51%) were male, and 18% were first-order births. The great majority of mothers were married (95%), age 20–49 (90%), and not working (72%). About three-fourths of mothers (74%) had normal body mass index (BMI). The large majority of households (86%) were male-headed, and 89% were in the rural areas of Ethiopia. By region, over three-quarters of children were located in just three regions: 19% in Amhara; 44% in Oromia; and 21% in SNNP. Nearly half of households (46%) were in the poorest or poorer wealth quintiles, versus a third (33%) in the richer or richest quintiles.

**Table 1:** Percent distribution of sampled children age 0–59 months by child’s, mother’s, and household characteristics, Ethiopia DHS 2016

Variables	N (%)
Child’s characteristics	
Age	
Less than 6 months	703 (7.5)
6–11 months	1018 (10.8)
12–17 months	1105 (11.7)
18–23 months	831 (8.8)
24–59 months	5762 (61)
Sex	
Male	4832 (51.3)
Female	4587 (48.7)
Birth order	
First	1656 (17.6)
2 to 4	4066 (43.2)
5 or higher	3697 (39.3)
Perceived weight at birth	
Very small	1474 (15.8)
Smaller than average	918 (9.8)
Average	3894 (41.6)
Very large or larger than average	3069 (32.8)
Mother’s characteristics	
Marital status	
Never married	53 (0.6)
Married/living with partner	8932 (94.8)
Widowed/divorced/separated	434 (4.6)
Age at child’s birth	
12–19	993 (10.5)
20–49	8427 (89.5)
Educational status	
No education	6251 (64.6)
Primary	2500 (26.5)
Secondary and above	668 (7.1)
Working status	
No	6780 (72.0)
Yes	2640 (28.0)
Mother’s BMI	
Underweight	1809 (19.7)
Normal	6774 (73.8)
Overweight	596 (6.5)
Maternal stature	
Normal stature	8932 (97.3)
Short stature	252 (2.7)
Household characteristics	
Sex of household head	
Male	8137 (86.4)
Female	1282 (13.6)
Place of residence	
Urban	1080 (11.5)
Rural	8339 (88.5)
Region	
Tigray	620 (6.6)
Afar	94 (1.0)
Amhara	1774 (18.8)
Oromia	4090 (43.4)
Somali	426 (4.5)
Benishangul	100 (1.1)
SNNP	2016 (21.4)
Gambela	23 (0.2)
Harari	22 (0.2)
Addis Ababa	215 (2.3)
Dire Dawa	39 (0.4)
Wealth quintile	
Poorest	2237 (23.7)
Poorer	2111 (22.4)
Middle	1984 (21.1)
Richer	1700 (18.0)
Richest	1387 (14.7)
Drinking water source	
Improved water	5347 (56.8)
Non-improved water	4072 (43.2)
Type of toilet	
Improved toilet	976 (10.4)
Non-improved toilet	8443 (89.6)
Type of cooking fuel	
Modern fuel	324 (3.4)
Traditional fuel	9095 (96.6)
Total	9419 (100)

### Bivariate associations of stunting and wasting by background characteristics

Table 2 indicates results from the bivariate analyses. Characteristics significantly associated with stunting included child’s age, sex, birth order, perceived weight, maternal education, mother’s BMI, place of residence, region, wealth quintile, toilet type, and fuel type. Variables associated with wasting included child’s age, perceived weight, mother’s BMI, region, wealth quintile, and fuel type.

**Table 2:** Bivariate analysis of stunting and wasting by child’s, mother’s, and household characteristics

Independent Variables	Outcome variables					
	Stunting %	95% CI	Chi2 *p*-value	Wasting %	95% CI	Chi2 *p*-value
Children’s characteristics						
Age			< 0.001			< 0.001
Less than 6 months	13.6	[10.0,18.3]		15.5	[11.5,20.5]	
6–11 months	17.2	[13.4,21.7]		12.9	[10.1,16.4]	
12–17 months	31.7	[27.7,36.1]		15.4	[12.3,19]	
18–23 months	48.6	[43.2,54.1]		11.3	[7.8,16.0]	
24–59 months	45.5	[42.9,48.1]		7.5	[6.5,8.6]	
Sex			< 0.001			0.760
Male	41.2	[38.7,43.8]		10.1	[8.8,11.6]	
Female	35.7	[33.4,38.2]		9.8	[8.4,11.4]	
Birth order			0.039			0.170
First	36.1	[31.8,40.7]		8.3	[6.4,10.7]	
2 to 4	37.0	[34.4,39.7]		9.7	[8.2,11.5]	
5 or higher	41.3	[38.5,44.3]		11.0	[9.5,12.7]	
Perceived weight at birth			< 0.001			0.002
Very small	46.2	[41.5,51]		13.4	[10.9,16.5]	
Smaller than average	43.6	[38.7,48.6]		12.0	[9.2,15.6]	
Average	37.6	[34.9,40.3]		9.6	[8.2,11.2]	
Very large or larger than average	34.6	[31.8,37.5]		8.2	[6.8,9.9]	
Mother’s characteristics						
Marital status			0.771			0.531
Never married	44.5	[26.8,63.8]		5.2	[0.8,28.1]	
Married/living with partner	38.4	[36.5,40.4]		9.9	[8.9,11]	
Widowed/divorced/ separated	40.6	[33.2,48.4]		12.0	[7.8,17.9]	
Age at child’s birth			0.111			0.478
12–19	42.5	[37.2,48]		9.0	[6.7,12]	
20–49	38.1	[36.1,40.5]		10.1	[9.0,12.2]	
Educational status			< 0.001			0.051
No education	41.7	[39.5,44]		10.8	[9.5,12.2]	
Primary	35.6	[32.5,38.8]		8.7	[7.1,10.7]	
Secondary and above	19.5	[15.3,24.4]		7.2	[4.8,10.7]	
Working status			0.947			0.410
No	38.5	[36.3,40.8]		9.7	[8.5,11.0]	
Yes	38.6	[35.7,41.7]		10.7	[8.8,12.9]	
Mother’s BMI			< 0.001			< 0.001
Underweight	41.9	[38.5,45.5]		13.5	[11.3,16]	
Normal	38.9	[36.5,41.3]		9.5	[8.4,16]	
Overweight	24.4	[19.6,29.9]		4.6	[2.4,8.8]	
Maternal stature			< 0.001			0.396
Normal stature	38.1	[36.1,40.1]		9.9	[8.8,11.1]	
Short stature	54.8	[45.5,63.7]		12.6	[7.4,20.7]	
Household characteristics						
Sex of household head			0.220			0.287
Male	38.4	[36.4,40.6]		10.1	[9.0,11.4]	
Female	39.2	[35.7,42.8]		8.8	[7.0,11.1]	
Place of residence			< 0.001			0.509
Urban	26.0	[21.5,31.1]		9.1	[7.0,11.8]	
Rural	40.1	[38.1,42.2]		10.1	[9.0,11.3]	
Region			< 0.001			< 0.001
Tigray	39.5	[35.8,43.4]		11.5	[9.5,13.8]	
Afar	40.9	[36.5,45.5]		17.1	[13.4,21.6]	
Amhara	47.6	[43.8,51.4]		10.0	[8.0,12.5]	
Oromia	36.1	[32.8,39.5]		10.5	[8.8,12.6]	
Somali	26.5	[22.8,30.6]		22.2	[17.5,27.8]	
Benishangul	42.9	[38.2,47.6]		10.5	[7.8,14.0]	
SNNP	39.6	[35.7,43.6]		6.2	[4.8,8.0]	
Gambela	24.1	[20.3,28.4]		13.9	[10.4,18.4]	
Harari	32.1	[27.7,36.8]		10.7	[7.4,15.2]	
Addis Ababa	14.9	[11.1,19.7]		2.4	[1.3,4.6]	
Dire Dawa	40.9	[34.5,47.6]		10.2	[7.1,14.4]	
Wealth quintile			< 0.001			< 0.001
Poorest	45.6	[42.4,48.8]		13.8	[11.2,16.8]	
Poorer	42.5	[38.7,46.5]		9.6	[7.7,12.0]	
Middle	38.6	[34,43.4]		10.4	[8.2,13.1]	
Richer	34.8	[31,38.7]		6.8	[5.1,9.0]	
Richest	25.7	[22.1,29.7]		7.7	[5.7,10.2]	
Drinking water source			0.048			0.098
Improved water	37.0	[34.6,39.5]		9.2	[8.0,10.5]	
Non-Improved water	40.5	[37.8,43.3]		11.0	[9.3,12.8]	
Toilet facilities			< 0.001			0.222
Improved toilet	26.8	[22.4,31.7]		8.5	[6.5,11.1]	
Non-improved toilet	39.8	[37.8,42]		10.0	[9.0,11.3]	
Type of cooking fuel			< 0.001			0.049
Modern fuel	21.4	[14.9,29.9]		5.2	[2.6,10.2]	
Traditional fuel	39.1	[37.2,41.1]		10.1	[9.1,11.3]	

### Factors associated with stunting

Table 3 shows the results of the adjusted multiple logistic regressions for stunning and wasting. Child’s age had a significant association with stunting. Children in the three age groups between 12 and 59 months were significantly more likely to be stunted compared with children age 0–6 months, with the highest odds found among children age 18–23 months. Female children were less likely to be stunted than male children. Children perceived as very large or larger than average in weight at birth were less likely to be stunted compared with children perceived as very small. Children of mothers with secondary and above educational attainment were less likely to be stunted compared with children of mothers with no education.

**Table 3:** Prevalence of stunting and wasting among children age 0–59 months by children’s, mother’s, and household characteristics

Independent variables		Outcome variables	
		Stunting	Wasting
		AOR (95%CI)	AOR (95%CI)
Children’s characteristics			
Age	Less than 6 months	1	1
	6–11 months	1.34 (0.97–1.86)	1.10 (0.82–1.50)
	12–17 months	3.50*** (2.59–4.74)	1.13 (0.84–1.52)
	18–23 months	7.81*** (5.73–10.65)	0.87 (0.62–1.21)
	24–59 months	6.59*** (5.00–8.68)	0.56*** (0.43–0.73)
Sex	Male	1	1
	Female	0.82*** (0.74–0.90)	0.77*** (0.67–0.88)
Birth order	First	1	1
	2 to 4	1.02 (0.87–1.20)	1.02 (0.80–1.29)
	5 or higher	1.16 (0.97–1.39)	1.16 (0.90–1.50)
Perceived weight at birth	Very small	1	1
	Smaller than average	0.82* (0.67–0.99)	1.07 (0.83–1.37)
	Average	0.68*** (0.59–0.78)	0.71*** (0.59–0.86)
	Very large or larger than average	0.54*** (0.47–0.63)	0.60*** (0.49–0.74)
Mother’s characteristics			
Marital status	Never in union	1	1
	Married/living with partner	0.72 (0.38–1.39)	2.16 (0.51–9.14)
	Widowed/divorced/ separated	0.64 (0.32–1.26)	2.48 (0.57–10.76)
Age	12–19	1	1
	20–49	0.97 (0.81–1.16)	0.96 (0.74–1.25)
Educational status	No education		
	Primary	0.97 (0.86–1.10)	0.92 (0.76–1.11)
	Secondary and above	0.66*** (0.52–0.84)	0.80 (0.57–1.13)
Working status	No	1	1
	Yes	1.02 (0.91–1.14)	1.08 (0.91–1.28)
Mother’s BMI	Underweight		
	Normal	0.84** (0.74–0.94)	0.67*** (0.57–0.78)
	Overweight	0.57*** (0.45–0.71)	0.39*** (0.28–0.55)
Maternal stature	Normal stature	1	1
	Short stature	2.03*** (1.44–2.86)	1.08 (0.65–1.80)
Households characteristics			
Sex of household head	Male	1	1
	Female	1.07 (0.94–1.23)	0.97 (0.81–1.17)
Place of Residence	Urban	1	1
	Rural	0.86 (0.69–1.07)	0.61 (0.64–1.10)
Region	Tigray	1	1
	Afar	0.87 (0.69–1.10)	1.33 (0.97–1.81)
	Amhara	1.27* (1.03–1.58)	0.87 (0.62–1.20)
	Oromia	0.83 (0.68–1.01)	1.00 (0.75–1.34)
	Somali	0.52*** (0.42–0.65)	2.02*** (1.51–2.68)
	Benishangul	1.13 (0.90–1.43)	0.97 (0.69–1.37)
	SNNP	0.99 (0.80–1.21)	0.60** (0.43–0.85)
	Gambela	0.51*** (0.39–0.66)	1.19 (0.84–1.67)
	Harari	0.87 (0.67–1.15)	1.20 (0.81–1.76)
	Addis Ababa	0.64* (0.43–0.94)	0.35** (0.17–0.76)
	Dire Dawa	1.09 (0.83–1.43)	0.83 (0.55–1.27)
Wealth quintile	Poorest	1	1
	Poorer	1.01 (0.86–1.17)	0.88 (0.71–1.10)
	Middle	0.77** (0.65–0.91)	0.92 (0.72–1.16)
	Richer	0.69*** (0.58–0.82)	0.66** (0.50–0.87)
	Richest	0.62*** (0.49–0.79)	0.58** (0.40–0.83)
Drinking water source	Improved water	1	1
	Non-improved water	0.91 (0.81–1.01)	1.00 (0.86–1.17)
Type of toilet facilities	Improved toilet	1	1
	Non-improved toilet	1.28** (1.07–1.53)	1.06 (0.83–1.36)
Type of cooking fuel	Modern fuel	1	1
	Traditional fuel	1.66** (1.18–2.32)	1.28 (0.74–2.21)

*** *p* < 0.001, ** *p* < 0.01, * *p* < 0.05, AOR: adjusted odds ratio

Children born of overweight and normal-BMI mothers were significantly less likely to be stunted compared with children of underweight mothers. Children of short-stature mothers were more likely to be stunted compared with children of not-short-stature mothers. There was significant regional variation in stunting among children under age 5. Children from Amhara region were more likely to be stunted compared with Tigray region. In contrast, children from Addis Ababa, Gambella, and Somali regions were less likely to be stunted. Children from the richest, richer, and middle household wealth quintiles were less likely to be stunted compared with children from the poorer and poorest quintiles. Children in households with non-improved toilet facilities were more likely to be stunted compared with those with improved toilet facilities. Children in households with traditional cooking fuel type were more likely to be stunted compared with those with modern fuel type.

### Factors associated with wasting

Table 3 also presents results of the adjusted multiple logistic regression for wasting. Children age 24–59 months were significantly less likely to be wasted compared with children age 0–6 months. Children with larger than average weight at birth and children with average perceived weight at birth were less likely to be wasted compared with children with perceived as very small at birth. Female children were less likely to be wasted than male children. Children whose mothers were overweight or of normal BMI had lower odds of being wasted compared with children of underweight mothers. Rural children were less likely to be wasted compared with urban children. Children in households in Addis Ababa and SNNP had lower odds of being wasted compared with children in Tigray region, while children in households in Somali region had higher odds of being wasted.

## Discussion

This study assessed the determinants of nutritional status among children under age 5 in Ethiopia. Study results show that child’s age, sex, and perceived birth weight, mother’s educational status, BMI, and maternal stature, and region, wealth quintile, type of toilet facility, and type of cooking fuel had significant associations with stunting. Child’s age, sex, and perceived birth weight, mother’s BMI, and residence and region showed significant associations with wasting.

This study shows that the risk of stunting increased with age of child. Previous studies in Ethiopia have shown similar results [22–24]. The reason might be that as children grow older they have greater energy needs. Besides, stunting reflects chronic malnutrition that can be manifested after long-term nutritional deficiency, while wasting reflects acute undernutrition. As child’s age increase, there is the probability that the child will receive childhood vaccinations, which reduce exposure to disease. For example [25] found that children vaccinated against measles are less likely to be wasted.

In Ethiopia as in many developing countries, complementary feeding is a challenge for children age 6–23 months [26]. Studies in other developing countries [26–28] found that complementary foods given to infants in the second 6 months of age and beyond are often inadequate in energy density.

In our study, female children were less likely to be stunted and wasted than boys. This finding is consistent with a meta-analysis in sub-Saharan Africa [16], a study in the Northern Ethiopia [29], and research in Myanmar [30]. However, it is contrary to findings of studies in Tanzania [31], Pakistan [32], India [33], and Kenya [34] that girls had a higher prevalence of stunting than boys. Example [16, 35, 36] found that sex differences in nutritional status might be due to biological differences in morbidity between boys and girls in their early life. This may also apply to our findings on wasting. A review of complementary feeding reported that boys have higher birth weights than girls and grow faster during infancy, resulting in greater energy needs [36]. Furthermore, another study on Guatemalan infants reported that women perceived male children to be hungrier and less likely to be satisfied by breastfeeding alone [37], placing them at increased risk of infections.

Regarding our findings on perceived weight of child at birth, the risks of stunting and wasting were higher among children perceived to have low birth weight. These findings are consistent with previous studies in Zambia [38], Bangladesh [39–41], India [42], Malaysia [43], and Indonesia [44], and may be due to the poor nutrition of mothers during pregnancy. This finding is supported by our results of the association between mother’s BMI and stunting and wasting in children. Ramakrishnan [45] reported that children born of overweight mothers are less likely to be stunted and wasted due to adequacy of protein and energy intakes of mothers during pregnancy [45]. A similar study in Nepal [46] also showed that as maternal BMI increased there was a reduction in children’s stunting, and wasting.

Our study found that children born to mothers with short stature were more likely to be stunted. This result is in line with previous studies in South Africa [14]; and Ghana [15]. This association reflects the fact that women who were stunted during their own childhood are more likely to give birth to stunted children [47].

Higher level of mothers’ educational attainment was positively associated with stunting in children. This is in line with previous related studies in the Philippines [48], Libya [49], Ethiopia [24], Uganda [50], Mozambique [51], and Ghana [15]. As stated in different studies, the reasons include that educated women are better informed about optimal child care practices [52], have better hygiene practices [53, 54], feeding [55], and childcare during illness [55–57], have a greater ability to use the health system [58], and are more empowered to make decisions [53]. In our study, however, in about two-thirds of cases (65%), the mothers had not attended formal education, and as a result their knowledge on childcare may not have been enough.

Our study found regional variation in stunting and wasting in Ethiopia. Compared with Tigray, the reference region, children from Addis Ababa were less likely to be either stunted or wasted, while children in Gambella were less likely to be stunted, and children in SNNP were less likely to be wasted. Interestingly, children in the Somali region were less likely to be stunted but more likely to be wasted compared with Tigray. This could be because wasting is characterized by acute malnutrition that can be caused by temporary increased food insecurity from extreme weather events, drought, and shifts in agricultural practices [59, 60]. Previous research in Ethiopia [61] showed the association between malnutrition varied across regions due to variation in cultivated area.

We found that children from the richest, richer, and middle household wealth quintiles were less likely to be stunted compared with children from the poorer and poorest quintiles. As mentioned, in the DHS surveys household wealth is measured as an index reflecting household material living standards, which include the types of water sources and sanitation facilities used by the household. Our study found that children in households with non-improved toilet facilities and traditional fuel types were more likely to be stunted. Previous studies in Nepal [62] and other Asian countries [63] have found that household asset accumulation is an important predictor of nutritional improvement.

Environmental factors such as unimproved water, unimproved sanitation, and biomass fuel are the second largest global attributable burden as cause of child stunting [64]. A study of 137 developing countries reported that an estimated 7.2 million cases of stunting were attributable to unimproved sanitation [64]. In Ethiopia, more than half of rural households (56%) use unimproved toilet facilities [9]. In rural areas of Ethiopia, more than one in three households (39%) have no toilet facility, versus 7% in urban areas [9]. Related studies in Sri Lanka [65–68] revealed that malnutrition among children is still a major health problem associated with poor sanitation and personal hygiene. However, a study conducted in rural Zimbabwe [69] found improved water, sanitation, and hygiene interventions in rural areas in low-income countries were unlikely to reduce stunting more than the effect of improved infant and child feeding alone. This implies integration of improved infant diets with improved water, sanitation, and hygiene is a logical approach to improve nutritional status of under-five children.

The great majority of Ethiopian households (93%) use traditional fuels such as wood, charcoal, and dung cake to meet their daily needs, and most households (59%) cook food outside [10]. Indoor air pollution and smoking are causes of low birth weight and could possibly impact stunting [45]. Another study in India reported associations between air pollution and stunting [70]; children living in households using biofuel were more likely to be stunted compared with children living in households using cleaner fuels ([70]).

## Strengths and limitations

The strength of this study was that it used nationally representative sample from urban and rural settings of the nine regional states and two urban administrations which allow generalization of the results. Therefore, these study findings could help as an intervention to policy makers, charity organizations across the globe for places having similar settings. It also applied all of the DHS data principles, like weighting. Regarding limitations, the cross-sectional data cannot examine causation or seasonal variation of nutritional outcomes. The other limitation is that during DHS data collection measurements of children’s height obtained by laying down children under age 2 may considered as a source of human error.

## Conclusion and policy implications

Our study found that child, maternal, and household characteristics were significantly associated with stunting and wasting among of children under age 5. Despite efforts made by the Ethiopian government and improvements in reducing malnutrition, rates of stunting and wasting remain high. The consequences of child malnutrition are grave and include poor performance in school and low adult economic productivity. The findings imply that a multi-sectorial and multidimensional approach is important to address malnutrition in Ethiopia. The education sector should promote maternal education and policies to reduce cultural and gender barriers. The health sector should provide health education to families to encourage positive behaviors toward childcare and infant feeding practices. In addition, the health and human services sectors can promote use of improved toilets. To reduce stunting due to traditional fuel usage in households, the energy sector should help households to access clean energy. Further research is suggested to explore the impact of seasonal food insecurity and climatic events on stunting and wasting and to examine the associated factors affecting nutritional status among children over time, using decomposition analysis.

## Data Availability

The weighted data used for the analysis can be obtained from the corresponding author up on reasonable request.
